# The impact of HBx protein on mitochondrial dynamics and associated signaling pathways strongly depends on the hepatitis B virus genotype

**DOI:** 10.1128/jvi.00424-24

**Published:** 2024-04-17

**Authors:** Anja Schollmeier, Michael Basic, Mirco Glitscher, Eberhard Hildt

**Affiliations:** 1 Division of Virology, Paul Ehrlich Institute, Langen, Germany; University of Southern California, Los Angeles, California, USA

**Keywords:** hepatitis B virus, HBx protein, genotypes, mitochondria, mitophagy, host response, reactive oxygen species

## Abstract

Chronic hepatitis B virus (HBV) infections are strongly associated with liver cirrhosis, inflammation, and hepatocellular carcinoma. In this context, the viral HBx protein is considered as a major factor influencing HBV-associated pathogenesis through deregulation of multiple cellular signaling pathways and is therefore a potential target for prognostic and therapeutic applications. However, HBV-associated pathogenesis differs significantly between genotypes, with the relevant factors and in particular the contribution of the genetic diversity of HBx being largely unknown. To address this question, we studied the specific genotype-dependent impact of HBx on cellular signaling pathways, focusing in particular on morphological and functional parameters of mitochondria. To exclusively investigate the impact of HBx of different genotypes on integrity and function of mitochondria in the absence of additional viral factors, we overexpressed HBx in Huh7 or HepG2 cells. Key signaling pathways were profiled by kinome analysis and correlated with expression levels of mitochondrial and pathogenic markers. Conclusively, HBx of genotypes A and G caused strong disruption of mitochondrial morphology alongside an induction of PTEN-induced putative kinase 1/Parkin-mediated mitophagy. These effects were only moderately dysregulated by genotypes B and E, whereas genotypes C and D exhibit an intermediate effect in this regard. Accordingly, changes in mitochondrial membrane potential and elevated reactive oxygen species production were associated with the HBx-mediated dysfunction among different genotypes. Also, genotype-related differences in mitophagy induction were identified and indicated that HBx-mediated changes in the mitochondria morphology and function strongly depend on the genotype. This indicates a relevant role of HBx in the process of genotype-dependent liver pathogenesis of HBV infections and reveals underlying mechanisms.

**IMPORTANCE:**

The hepatitis B virus is the main cause of chronic liver disease worldwide and differs in terms of pathogenesis and clinical outcome among the different genotypes. Furthermore, the viral HBx protein is a known factor in the progression of liver injury by inducing aberrant mitochondrial structures and functions. Consequently, the selective removal of dysfunctional mitochondria is essential to maintain overall cellular homeostasis and cell survival. Consistent with the intergenotypic difference of HBV, our data reveal significant differences regarding the impact of HBx of different genotypes on mitochondrial dynamic and function and thereby on radical oxygen stress levels within the cell. We subsequently observed that the induction of mitophagy differs significantly across the heterogenetic HBx proteins. Therefore, this study provides evidence that HBx-mediated changes in the mitochondria dynamics and functionality strongly depend on the genotype of HBx. This highlights an important contribution of HBx in the process of genotype-dependent liver pathogenesis.

## INTRODUCTION

Chronic hepatitis B virus (HBV) infection remains a major global health problem, estimated to affect 296 million carriers worldwide. It is a leading cause for infectious diseases and contributes to cancer-related deaths (1, 2). A persistent, chronic infection is characterized by different phases among disease progression and leads to the development of end-stage liver disease, hallmarked by increasing hepatic inflammation, liver cirrhosis, and hepatocellular disease (3–5).

Moreover, the pathogenesis and clinical outcome tremendously differ with respect to the HBV genotype (6). Traditionally, HBV genotypes were associated with distinct geographical distributions, classified by a divergence in nucleic acid sequences of greater than 8% across the entire HBV genome, due to error-prone replication (7). In addition, treatment response, hepatocellular carcinoma (HCC) development, hepatitis B surface antigen (HBsAg) seroclearance, hepatitis B e antigen (HBeAg) seroconversion, as well as several virological and cell-biological properties, differ across the genotypes. Therefore, they become an increasing focus in current HBV research (8–11).

Phylogenetically, HBV has been classified into nine genotypes (A–I) and a putative 10th genotype J (12, 13). While the genotypes A and C display a higher tendency of chronicity, patients infected with genotypes C, D, and F display a lower or delayed rate of anti-HBe seroconversion. Among chronic carriers, genotypes C, D, and F are associated with a rapid disease progression with HCC development and therefore show a poor clinical outcome (14, 15). Besides this, genotype G behaves as aberrant HBV genotype without a clear prevalence and is frequently detected as co-infection with other HBV genotypes or the human immunodeficiency virus type 1 (16, 17). As genotype G fails to secrete HBsAg and HBeAg, this could lead to an underdiagnosis (9). Notably, elevated inflammation processes and liver pathogenesis are driven by multiple factors being regulated by viral proteins (18).

The partially double-stranded HBV genome contains four overlapping open reading frames (ORFs; Pol-, preS/S-, preC/C- and X-ORF) coding for seven viral proteins (19). Among these, the smallest ORF encodes for the 154 amino acid regulatory HBx protein with a molecular weight of 17 kDa (20). However, the lack of a known structure and the tendency to aggregate within cells, together with a low expression level upon HBV infection, hamper the study of fundamental biological properties of HBx. Also, HBx studies in the context of natural HBV infection represent a major challenge because of limited and inefficient cell culture-based infection systems and the absence of specific and sensitive HBx antibodies (21). Nevertheless, because of the pleiotropic effects on several host signaling pathways and dysregulation of oncogenic modulators, HBx is discussed as the driving force in the progression of chronic liver disease (18, 22). However, there is no detailed comparative analysis of HBx from different genotypes on mitochondrial dynamics and function.

In particular, a proportion of HBx interacts with host-cell mitochondria and profoundly modulates mitochondrial homeostasis and network morphology (23, 24). In general, mitochondria represent a central part in regulating metabolic and signal transduction pathways to maintain cellular homeostasis, metabolism, and immune signaling. In this pursuit, mitochondria coordinate a highly dynamic process of fission, fusion, and organelle-selective autophagy (mitophagy) to maintain mitochondrial function and overall cell homeostasis (25).

The viral HBx protein, however, shifts the mitochondrial network dynamics toward an elevated, aberrant mitochondrial fragmentation (fission) and subsequent mitophagy (26). In this regard, HBx was described to trigger an induction of mitophagy by hijacking PTEN-induced putative kinase 1 (PINK1) with subsequent activation of the PINK1/Parkin-mediated pathway (27, 28). In addition, the interaction of HBx with the voltage-dependent anion channel 3 (VDAC3) is reported as causative factor for changes in the mitochondrial membrane potential with further consequences for the mitochondrial respiratory chain, as well as release of cytochrome c and reactive oxygen species (ROS) (29, 30). The release of mitochondrial metabolites, upon membrane depolarization, is connected with an aberrant regulation of antioxidant genes and increase of cytokines as inflammatory mediators (31). Furthermore, there is an intimate crosstalk between increased cellular ROS levels and mitochondrial dysfunction and damage (32).

Overall, it is hypothesized that HBx utilizes mitochondrial function and mitophagy as a conductive factor for cell survival and hence viral persistence, although mitochondrial dysfunction and oxidative stress are directly associated with serious impact in chronic liver disease and HCC development (25). However, the consequences of genetic variability among HBV genotypes on HBx function have not been extensively studied.

This work aims to broaden the current understanding of the HBV genotype-related impact of the HBx protein, with particular focus on morphological and functional implications on mitochondria dynamics and the interaction with associated host proteins. To exclusively study the impact of HBx of different genotypes on the integrity and function of mitochondria in the absence of additional viral factors, we overexpressed HBx in Huh7 or HepG2 cells and analyzed the HBV genotype-dependent impact of HBx on mitochondrial dynamics and function. We found that the impact of HBx on these parameters strongly depends on the genotype. In addition, in the context of a full-length HBV replicating system, we could verify aberrant mitochondrial structures dependent on different HBV-genotypes. Overall, this reflects the relevance of HBx for the process of genotype-dependent liver pathogenesis.

## RESULTS

### Characterization and validation of the study approach

**Fig 1:**
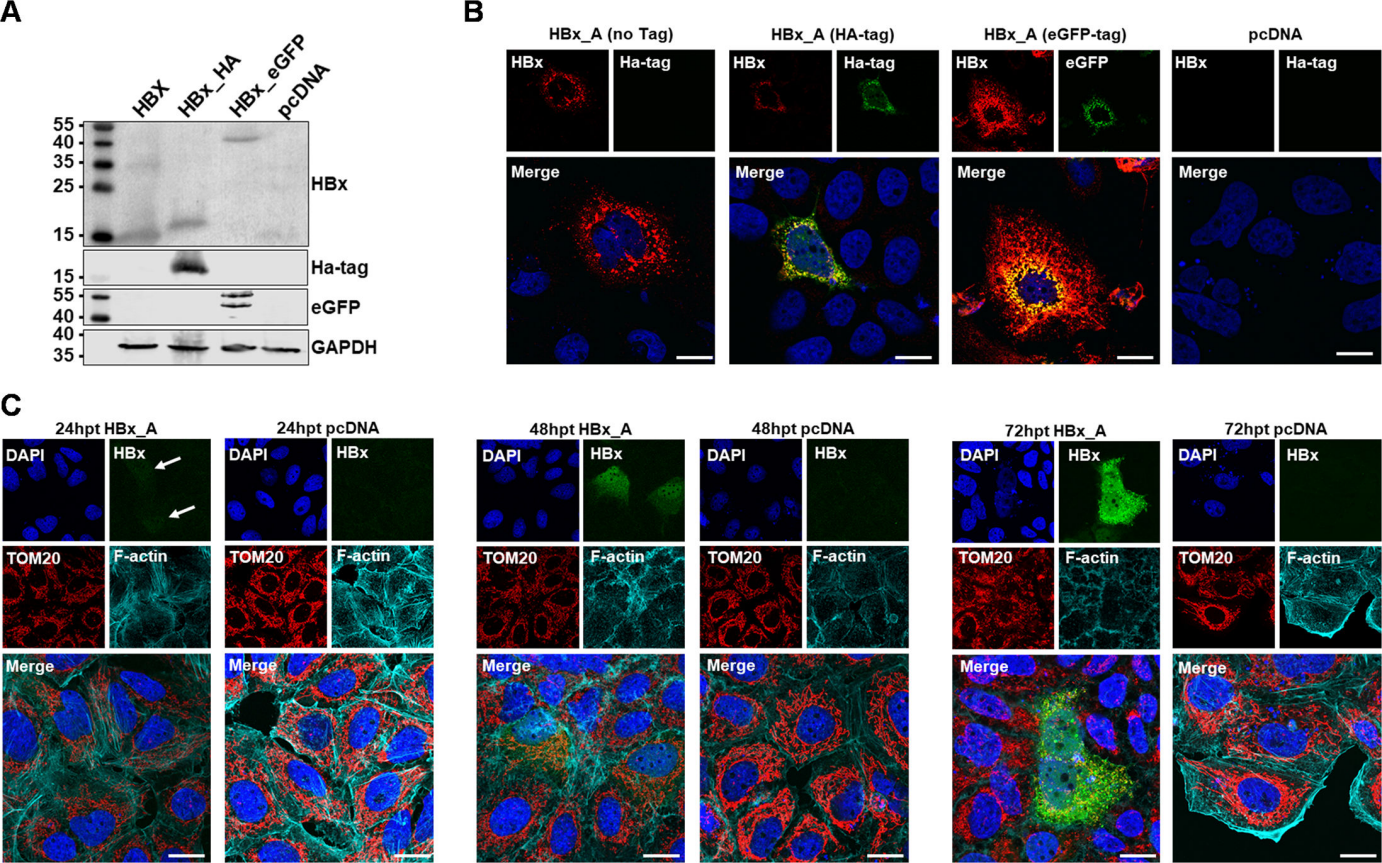
Characterization of HBx constructs. Huh7 cells were transfected with HBx_gtA (no tag), HBx_gtA-HA, HBx_gtA-eGFP or empty plasmid DNA as control. (**A**) Representative Western blot of cell lysate [72 h post transfection (pt)] proceed for HBx protein expression using a HBx-specific antibody. Specific tags were detected by eGFP or HA-specific antibodies. β-actin was used as loading control. (**B**) Representative immunofluorescence staining of HBx (red), HA-tag/eGFP (green), and nuclei (blue). Scale bar indicates 20 µm. (**C**) Representative confocal microscopy images of HBx_gtA-Ha-transfected cells, harvested 24, 48, or 72 h post transfection. Cells were immunostained for HBx with HA-specific antibody (green) and TOM20 antibody (red) to image the outer mitochondrial membrane. F-actin was counterstained with phalloidin-Atto 633, nuclei with 4′,6-diamidin-2-phenylindole (DAPI) (blue). Scale bar indicates 20 µm. White arrows indicate HBx staining at very low-expression levels.

In order to characterize HBx-specific effects in the absence of any interference by other viral (regulatory) proteins, an experimental system based on exclusive overexpression of HBx was chosen. To avoid a bias based on differences in the detectability by HBx-specific antibodies due to genotype-dependent variability in the epitopes recognized by the antibodies, hemagglutinin (HA)-tagged or enhanced green fluorescent protein (eGFP)-tagged constructs were instrumental. As all constructs share the same tag for detection, a variability due to differences in the recognition by antibodies can be excluded. The expression of HBx and the respective HA or eGFP tag and the comparability of the respective constructs were confirmed by Western blot analysis of cellular lysates derived from Huh7 cells transiently transfected with HBx-GtA either without tag or with the above described HA or eGFP tag (Fig. 1A). In addition, based on confocal laser scanning microscopy (CLSM), immunostaining with an HBx-specific antibody and co-staining with the respective tag verified an equal localization and distribution among the different HBx constructs (Fig. 1B). Furthermore, the intracellular localization of HBx-GtA in Huh7 cells was determined at different time points [24, 48, and 72 h post transfection (p.t.)]. At low expression levels (24 h p.t.), HBx localizes exclusively in the nucleus and was only barely detectable by immunofluorescence microscopy (white arrow, Fig. 1C). After 48 h, HBx is still mainly present in the nucleus; however, slight amounts are also visible in the cytoplasm. At both time points, the mitochondrial network remains completely intact and is distributed over the whole cytoplasm and extends up to the outer cell membrane. At high expression levels after 72 h p.t. (reflects also a persistent infection) the HBx protein is distributed in the cytoplasm of the cell and is able to interact with mitochondrial proteins. The mitochondrial structure itself demonstrated in the presence of HBx at this late time point a condensed and perinuclear localization (Fig. 1C).

In summary, the tagged HBx constructs used in the present study express and localize the viral HBx protein in a similar manner, compared to the unlabeled HBx protein. In addition, the localization of HBx is time dependent and represents mainly cytoplasmic localization and therefore mitochondrial accessibility after 72 h p.t.

### Cellular distribution and amount of HBx significantly differ between the genotypes

**Fig 2:**
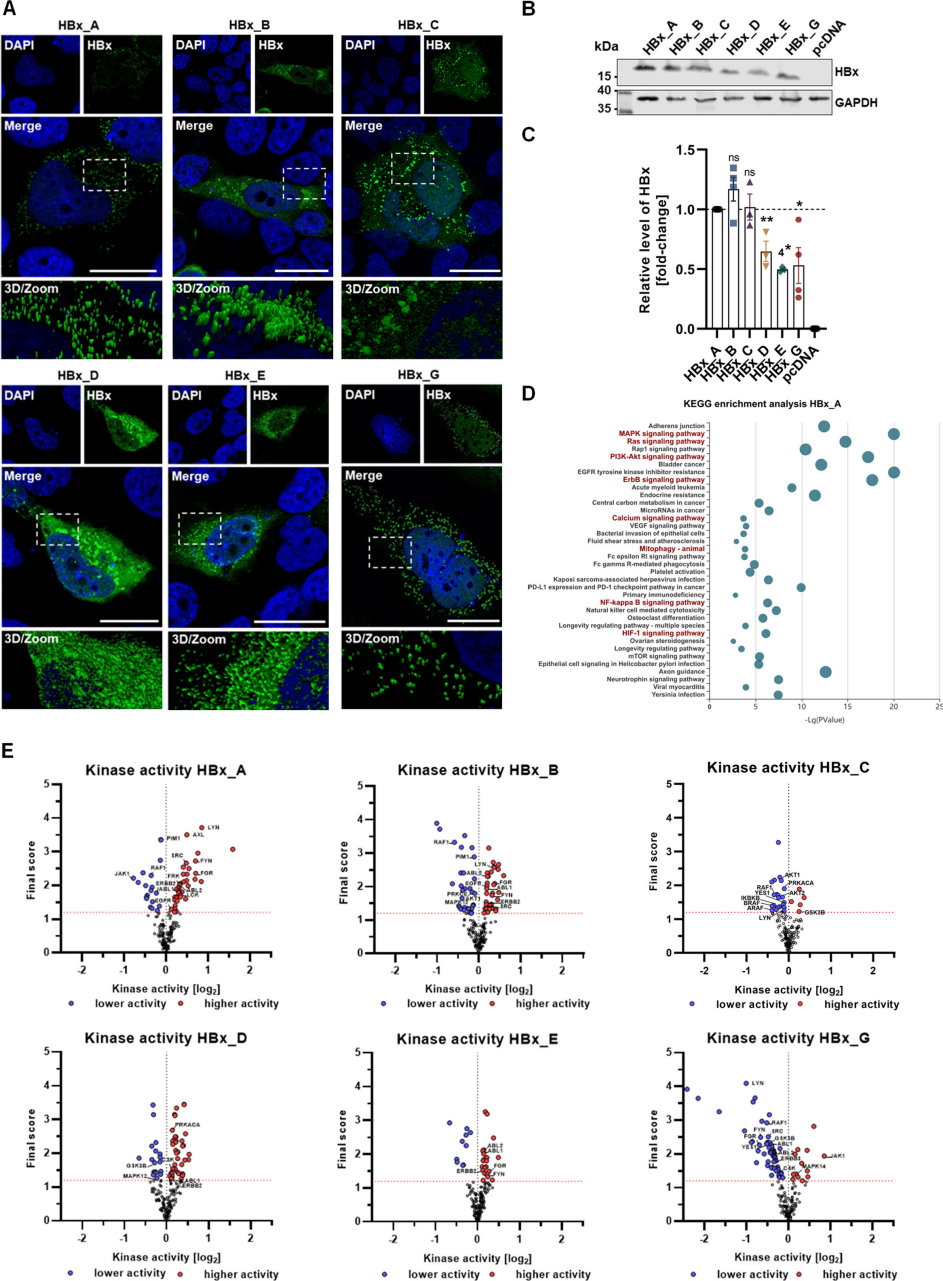
HBx protein of different genotypes displays distinct differences in subcellular distribution and kinome profile. Huh7 cells were transiently transfected with HBx plasmid DNA of either GtA, GtB, GtC, GtD, GtE, GtG or empty plasmid DNA as control, and fixed for immunofluorescence or harvested 72 h p.t. (**A**) Representative confocal microscopy images with three-dimensional high-resolution zoom sections, indicated by the dashed rectangles. Cells were immunostained for HBx with HA-specific antibody (green). Nuclei were counterstained with DAPI (blue). Scale bar indicates 20 µm. (**B**) Representative Western blot of cell lysates, processed for HBx protein levels using HA tag-specific antibody and glyceraldehyde-3-phosphate dehydrogenase as internal loading control. (**C**) Western blot quantification of panel B; relative HBx protein level was calculated based on *n* > 3 independent experiments. Data presented as mean ± standard error of the mean. Statistical analysis was performed using unpaired *t*-test related to the HBx_A sample. (**D**) Significantly enriched KEGG pathways by KOBAS with *P* value of >0.05. Potential predicted kinases with a mean final score of >1.2 were conducted for KEGG pathway enrichment analysis. Each bubble indicates an enriched KEGG term; the top five terms with the highest enriched ratio is displayed. The node size represents the *P* value of enrichment (from small to large): (0.05–1.0), (0.01–0.05), (0.001–0.01), (0.0001–0.001), (1e^−10^, 0.0001), (0, 1e^−10^), which means that bigger bubbles reflect smaller *P* values. (**E**) Volcano plot for predicted kinase activity. The red dots indicate significantly upregulated kinases; the blue dots represent significantly downregulated kinases. A mean final score above 1.2 (red line) was conducted to be significant. Kinases below a mean final score of 1.2 are colorless. Significantly activated kinases with known impairment in mitochondrial function were labeled by name. *P < 0.05, **P < 0.01. KEGG, Kyoto Encyclopedia of Genes and Genomes; KOBAS, KEGG Orthology Based Annotation System; ns, not significant.

To investigate the subcellular localization and distribution of HBx protein of different genotypes (Gt’s), Huh7 cells were transiently transfected with the respective HBx plasmid DNA and fixed 72 h p.t (Fig. 2A through C). In this regard, immunofluorescence staining of HBx demonstrates different subcellular distributions among HBV genotypes. We observed for GtA, GtC, and GtG a dot-like distribution of the HBx protein over the whole cytoplasm. In contrast, HBx of genotypes B, D, and E occurs with a homogenous distribution throughout the cytoplasm. However, a pronounced perinuclear accumulation in case of HBx-GtB and HBx-GtD is observed. Despite this, all genotypes showed a predominantly cytoplasmic localization with only weak nuclear signals for HBx (Fig. 2A). Analysis of the HBx amount by Western blot, reveals a higher amount for the HBx-GtA, GtB, and GtC as compared to the GtD, GtE, and GtG. In particular, in case of GtE, the level of HBx expression was on average reduced by 50% as compared to HBx-GtA. However, especially GtG and GtD have a high variance among the experimental replicates (Fig. 2B and E). Taken together, these data indicate for all investigated HBx variants an extra-nuclear distribution, but there are clear differences with respect to the cytosolic staining pattern.

### The impact of HBx on the kinome profile strongly depends on the genotype

To gain further understanding of the impact of the genotype on HBx function, a kinome analysis was performed using a phosphopeptide array-based method (Fig. 2D through E). This method facilitates the identification of kinome activity in order to investigate signal transduction and pathway alterations. In light of this, predicted targets of HBx-GtA-transfected Huh7 cells with a mean final score above 1.2 were used for Kyoto Encyclopedia of Genes and Genomes (KEGG) pathway enrichment. The most significantly enriched KEGG terms (*P* value of >0.05) were identified and categorized into different clusters. The top five enriched KEGG terms are displayed in Fig. 2D and includes central signal transduction pathways such as MAPK, Ras, PI3K signaling. Importantly, several enriched pathways are directly associated with pleiotropic effects on the mitochondrial biogenesis and related inflammatory response. These terms are highlighted red in Fig. 2D and includes mitophagy, ErbB signaling, calcium signaling, as well as NF-κB and HIF1 signaling. Furthermore, a comparison of putative enriched kinases among all genotypes was investigated, with particular focus on mitochondrial related kinases. In this regard, volcano plots for differentially enriched kinases of each HBx variant are displayed, with upregulated kinases indicated in red and downregulated kinases represented in blue (Fig. 2E). Based on these data, HBx protein of the used genotypes demonstrated biological functional activity for all variants. However, for GtA and GtB, a significantly higher upregulated kinase activity was found as compared to GtC and GtE, whereas in the case of GtG, the strongest level of downregulation was observed. With respect to kinases affecting mitochondria, Src kinase family, ABL, EGFR, ErbB2, Akt, JNK, ERK1/ERK2, p38 MAPK, GSK3β, PKA, and PKC were determined by Lim et al. as key kinases in mitochondria regulatory processes (33). As annotated in Fig. 2E, these kinases were labeled in the respective volcano blots of the different genotypes, if they were significantly deregulated. Based on this, for HBx-GtA and GtB, a significant upregulation of Scr family kinases, together with ABL kinases, but a downregulation of RAF1 and ErbB2 was found. In contrast, only few mitochondrial-related kinases were deregulated in HBx-GtD and GtE. For GtG, a downregulation of the Scr-kinase family, but a strong upregulation of JAK1 and MAPK14 was detected. In summary, the kinome profile of HBx strongly differs with respect to the genotype. This suggests different effects on the overall cellular function and in particular on mitochondrial kinases.

### HBx leads to profound changes in the mitochondrial structure with significant genotype-related differences

**Fig 3:**
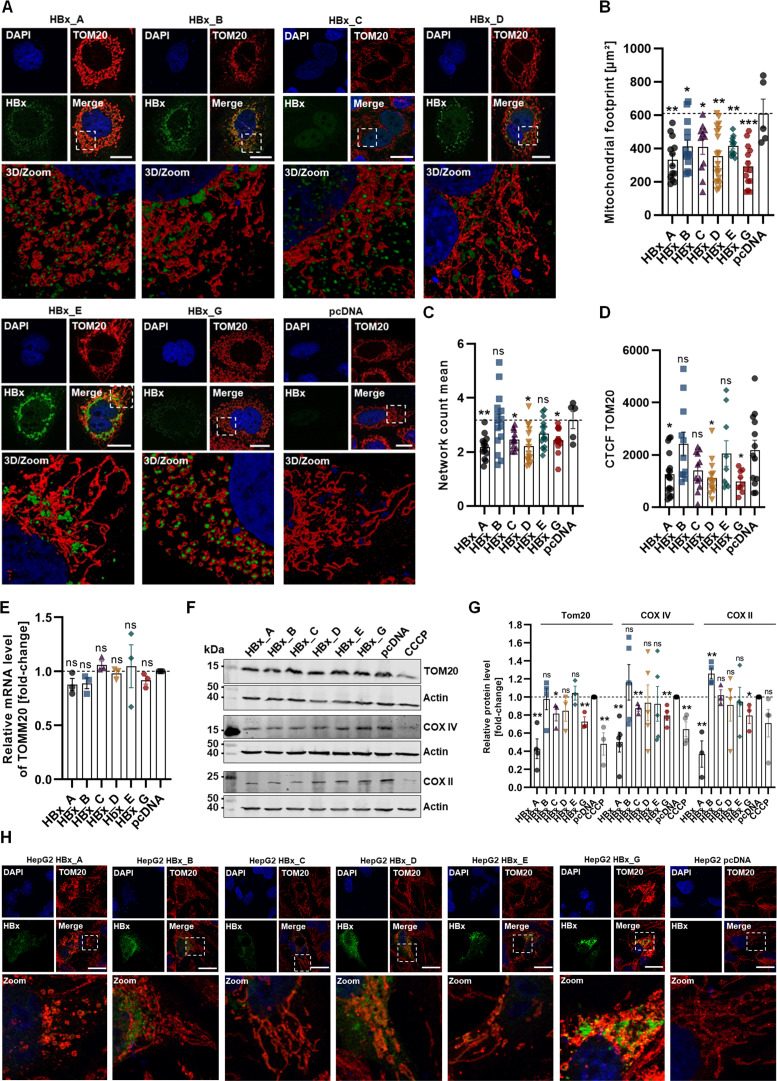
HBx induces alterations in the mitochondrial dynamics and network structure. Huh7 or HepG2 cells were transiently transfected with HBx plasmid DNA of either GtA, GtB, GtC, GtD, GtE, GtG or empty plasmid DNA as control, and fixed for immunofluorescence or harvested 72 h p.t. (**A**) Representative confocal microscopy images with 3D high-resolution zoom sections indicated by dashed rectangle. Huh7 cells were immunostained for HBx with HA-specific antibody (green) and TOM20 antibody (red) to image the outer mitochondrial membrane. Nuclei were counterstained with DAPI (blue). Scale bar indicates 20 µm. (**B**) Quantification of A; mitochondrial footprint indicates the area of mitochondrial pixels in squared micrometer. More than 12 cells per genotype, pcDNA *n* = 5, were analyzed. (**C**) Quantification of A; network count mean reflects the mean number of connected mitochondria. More than 12 cells per genotype, pcDNA *n* = 5, were analyzed. (**D**) Quantification of the corrected total cell fluorescence of TOM20 staining in panel A. A minimum of 10 cells were analyzed. (**E**) Huh7 mRNA levels of *TOM20*, determined by real-time quantitative PCR. Data represent the fold change as compared to pcDNA control from *n* = 3 independent experiments. (**F**) Representative Western blot of Huh7 cell lysates, processed for mitochondrial protein levels using TOM20, COX II, and COX IV-specific antibody. β-Actin protein level was used as internal loading control. CCCP, a mitochondrial decoupler, was used as a control to introduce mitochondrial degradation. (**G**) Western blot quantification of panel F; relative TOM20, COX II, and COX IV protein levels, based on *n* > 3 independent experiments. All data are indicated as mean ± standard error of the mean. Statistical calculation was performed using unpaired *t*-test related to the pcDNA sample. *P < 0.05, **P < 0.01, ***P < 0.001. (**H**) Representative confocal microscopy images with zoom sections indicated by dashed rectangle. HepG2 cells were immunostained for HBx with HA-specific antibody (green) and TOM20 antibody (red) to image the outer mitochondrial membrane. Nuclei were counterstained with DAPI (blue). Scale bar indicates 20 µm. 3D, three-dimensional; CCCP, carbonyl cyanide m-chlorophenyl hydrazine.

The previous data reflect changes in the mitochondrial kinome, together with a high heterogeneity among the genotypes of HBx. For a detailed analysis of the crosstalk between genotype of HBx and the mitochondrial network, mitochondria structures were stained via the marker protein TOM20 in Huh7 cells expressing HBx of different genotypes and analyzed by confocal microscopy with a high-resolution lightning tool. The amount of TOM20, as well as the respiratory chain component cytochrome c oxidase subunit II (COX II) and cytochrome c oxidase subunit IV (COX IV), was assessed via Western blotting in order to observe changes in the overall mitochondrial mass, as depicted in Fig. 3.

Based on the structural analysis of mitochondria, the control-transfected cells (empty vector plasmid) indicate the expected reticular network structure of mitochondria with highly elongated tubular filaments, whereas cells expressing HBx display a reduction in mitochondrial network and footprint. While this is only moderately pronounced for GtB and GtE, reflected by only slightly shorter tubular filaments and interconnections of the network, GtC and GtD display a certain amount of mitochondrial fragmentation. Strikingly, both GtA and GtG display heavily disrupted mitochondria represented by a swollen and round mitochondrial shape, without any branching to other mitochondria.

In this regard, the distribution of HBx protein appears closely related to the fragmented mitochondria. The more perinuclearly accumulated HBx protein in the other genotypes (GtB, GtC, GtD, and GtE) is located in between the mitochondrial networks without direct association. (Fig. 3A). A quantification of the mitochondrial network by using an ImageJ mitochondrial network analyzer tool confirms the previously observed investigations. All HBx proteins of the investigated genotypes induced a decline in the overall mitochondrial footprint but with tremendously lower levels for GtA and GtG (Fig. 3B). The mean network count indicates the average number of connected mitochondria and confirms a significant decline in the HBx-GtA-, GtD-, and GtG-expressing cells (Fig. 3C). Moreover, these observations are accompanied by a significant loss in the TOM20 protein level, which is not based on gene expression, as determined by calculation of corrected total cell fluorescence (CTCF), WB analyses, and real-time quantitative PCR (RT-qPCR) (Fig. 3D through F).

The amount of the nuclear-encoded mitochondrial proteins TOM20 (localized at the outer mitochondrial membrane) as well as COX IV and the mitochondrial-encoded COX II (localized at the inner mitochondrial membrane) was determined to observe if the overall mitochondrial mass is affected by the different genotypes of the HBx protein. Mitochondrial uncoupler-treated cells [carbonyl cyanide m-chlorophenyl hydrazine (CCCP)] were used as a control for induced mitochondrial damage. Consistent for all analyzed mitochondrial proteins, in case of HBx the GtA and GtG, a significant and up to 50% lower level was found as compared to the pcDNA-transfected control. HBx of GtC induced as well a significant decrease in the Tom20 and COX IV expression level compared to the control, but not for COX II. The other genotypes could not induce a significant decline in the level of the analyzed proteins compared to the control (Fig. 3G). To exclude any Huh7 cell line-specific effects, the experiments were performed in HepG2 cells in addition. Comparable observations were made for the HBx expression in the human hepatoma cell line HepG2. Based on immunofluorescence microscopy, again a strong fragmentation in the mitochondrial network is visible mainly for HBx-GtA and GtG. HBx-GtC and GtD have a lower amount of branched structures, whereas GtB and GtE retain the elongated tubular mitochondrial structures (Fig. 3H). Overall, we observed profound changes in the morphology and mass of mitochondria by HBx but with major differences among the genotypes. Importantly, changes in the mitochondrial structure are consistent among different human hepatoma cell lines.

### HBx genotypes differ with respect to their impact on mitochondrial membrane potential and homeostasis

**Fig 4:**
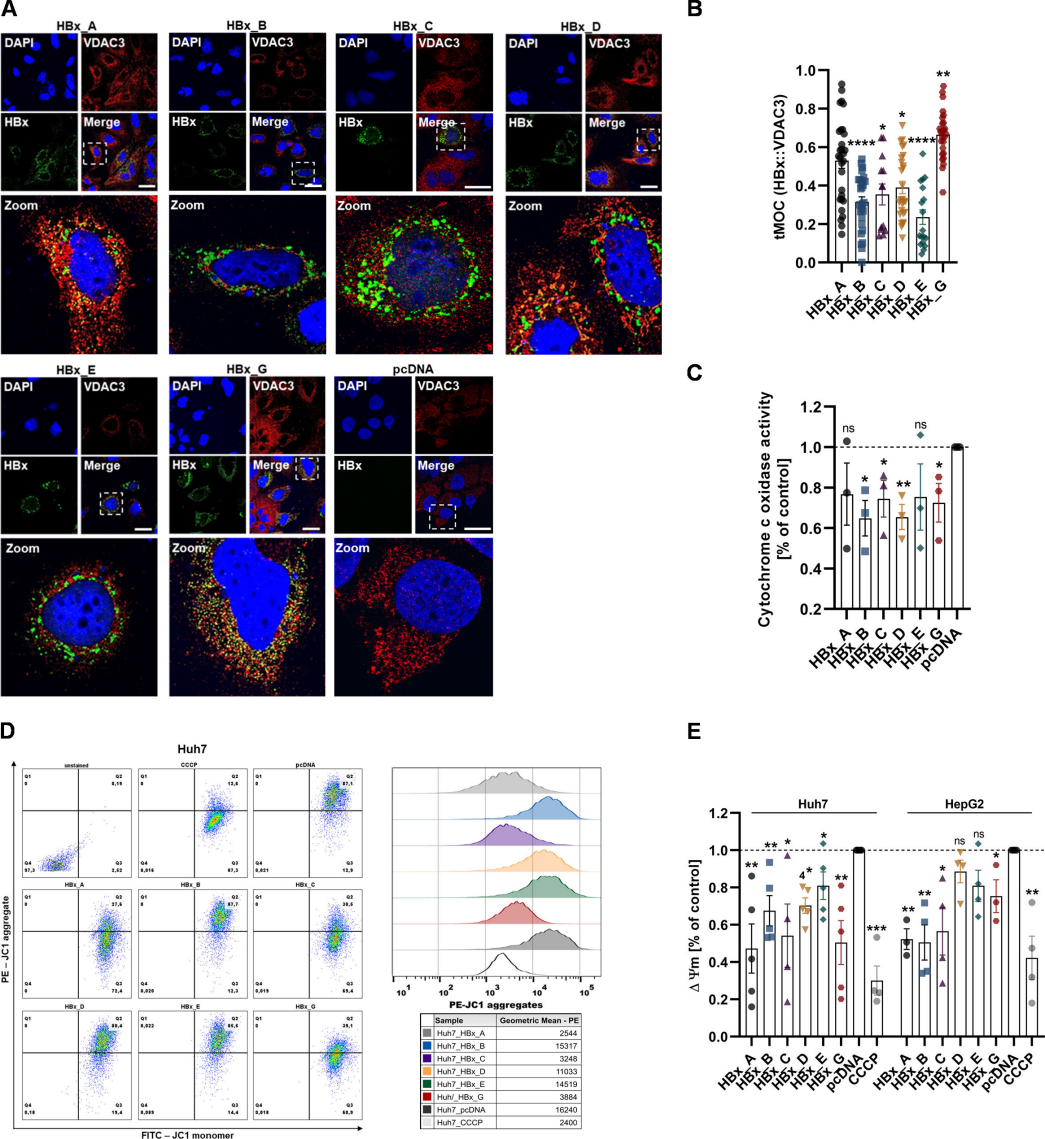
HBx influences mitochondrial homeostasis and function in a genotype-dependent manner. Huh7 or HepG2 cells were transiently transfected with HBx plasmid DNA of either GtA, GtB, GtC, GtD, GtE, GtG or empty plasmid DNA as control, and fixed for immunofluorescence or harvested 72 h p.t. (**A**) Representative confocal microscopy images with zoom sections indicated by dashed rectangles. Huh7 cells were immunostained for HBx with HA-specific antibody (green) and VDAC3-specific antibody (red). Nuclei were counterstained with DAPI (blue). Scale bar indicates 40 µm. (**B**) Quantification of panel A; tMOC of VDAC3 with HBx protein (green signals are present in red areas per cell). More than 18 cells per condition were analyzed. Statistical calculation was performed using unpaired *t*-test related to HBx_gtA sample. (**C**) Cytochrome c oxidase activity assay of Huh7 cells, calculated by the rate of change in the linear range and normalized to the pcDNA control sample. *n* = 3 independent experiments. (**D**) Flow cytometry analysis of HBx or pcDNA-transfected as well as CCCP-treated Huh7 cells were stained with the mitochondrial membrane-sensitive JC-1 dye. Representative dot blots represent JC-1 monomers in the fluorescein isothiocyante (FITC) channel on the *x*-axis; JC-1 aggregates are displayed on the *y*-axis of the PE channel as well as in the representative histogram with calculated geometric mean fluorescence intensities of phycoerythrin (PE). (**E**) Quantification of D as well as HepG2-transfected cells; the ratios of red (JC-1 aggregates) to green (JC-1 monomers) mean fluorescence intensities were calculated and indicate the final mitochondrial membrane potential relative to the pcDNA control of *n* > 3 independent experiments. All data are represented as mean ± standard error of the mean. As not indicated otherwise statistical calculation was performed using unpaired *t*-test related to pcDNA sample. *P < 0.05, **P < 0.01, ***P < 0.001, ****P < 0.0001. CCCP, carbonyl cyanide m-chlorophenyl hydrazine; tMOC, thresholded Mander’s overlap coefficient.

The high degree of HBx-mediated mitochondrial disruption, especially in case of GtA and GtG, suggested also a potential severe impact on the mitochondrial homeostasis and function. This may be manifested through the interaction with the ion-channel VDAC3, as evidenced by a previous study (29). Hence, to gain further insights in the genotype-dependent impact of HBx on the mitochondrial function, the interaction of VDAC3 with HBx was assessed by confocal microscopy in transiently transfected Huh7 cells. Flow cytometry with a membrane potential-sensitive dye, as well as a cytochrome c oxidase activity assay, was utilized to observe genotype-dependent alterations in the mitochondrial homeostasis (Fig. 4).

We assessed for all of the analyzed variants of HBx an interaction with the VDAC3; however, strong differences were observed in the level of colocalization. While the genotypes B and E occur with the lowest level of co-localization with an average thresholded Mander’s overlap coefficient (tMOC) value between 25% and 35%, the highest co-localization was found in the case of HBx of the genotypes A and G, with an average tMOC value between 55% and 70%. For HBx-GtC and GtD, a colocalization value (tMOC) around 40% was revealed (Fig. 4A and B). This was in line with the investigation of mitochondrial cytochrome c oxidase activity, as part of the respiratory chain complex. While an overall reduction in HBx expression could be observed, the change for the GtA and GtE was not significant. For HBx of genotypes B, C, D, and G (Fig. 3E), a significant decline in the activity was detected (Fig. 4C).

In accordance to this, flow cytometry experiments using the mitochondrial membrane potential-sensitive JC-1 dye clearly displayed a shift of the red JC-1 aggregate toward the green JC-1 monomer in the HBx-transfected samples compared to the control, indicating a loss of membrane potential (Fig. 4D). While this was less pronounced for GtB, GtD, and GtE, HBx-GtA, GtC, and GtG demonstrated a strong decline in the mitochondrial membrane potential with up to 42% reduction as compared to the control. Accordingly, also in the HepG2 cell line, the mitochondrial membrane potential declined in a genotype-dependent manner (Fig. 4E).

Taken together, these data indicate a strong and genotype-dependent impact of HBx on the mitochondrial function. Just as the impact on mitochondrial morphology, this effect is most pronounced for HBx derived from GtA and GtG.

### HBx-mediated perturbation of mitochondrial integrity induces oxidative stress and proinflammatory response in a genotype-dependent manner

**Fig 5:**
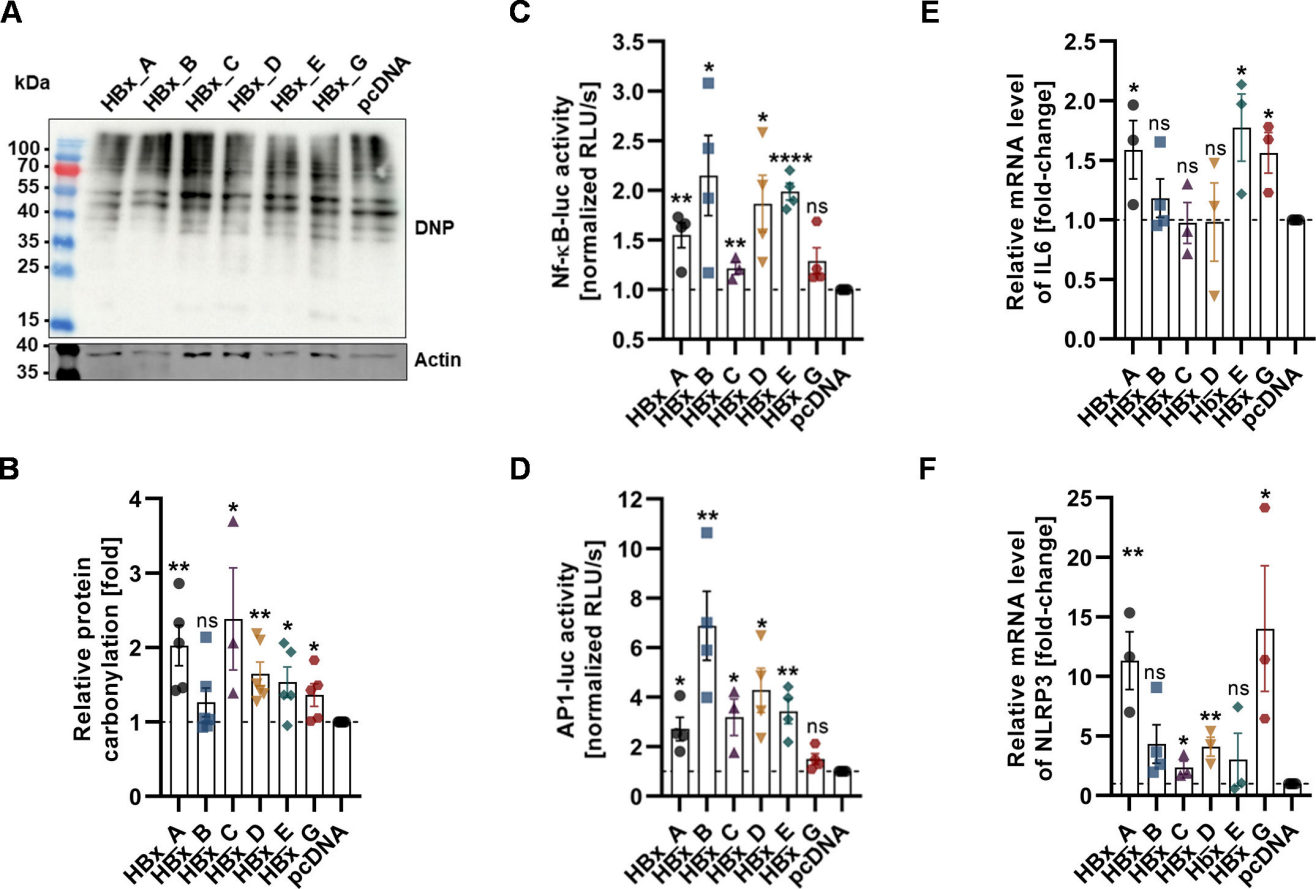
Disturbance of mitochondrial integrity by HBx induces ROS formation and related host signaling response. (**A**) Representative OxyBlot of whole cell lysates from Huh7 cells, transfected with HBx plasmid DNA of either GtA, GtB, GtC, GtD, GtE, GtG or empty plasmid DNA as control. The membrane was processed with anti-DNP antibody to detect derivatized carbonyl groups in the proteins and β-actin as internal loading control. (**B**) OxyBlot blot quantification; relative amount of DNP derivates normalized to the control sample was calculated based on *n* = 6 independent experiments. (**C**) Luciferase reporter gene assay, depicting the nuclear factor kappa B (Nf-кB) promoter activity of HBx co-transfected Huh7 cells, 48 h p.t. Data represent normalized RLU/s of luciferase activity compared to the control sample of *n* = 4 independent experiments. (**D**) Luciferase reporter gene assay, depicting AP-1 promoter activity of HBx co-transfected Huh7 cells, 48 h p.t. Data represent normalized RLU/s of luciferase activity compared to control sample, *n* = 4. (**E**) mRNA transcription levels of IL-6, determined by RT-qPCR. Data represent the fold change as compared to pcDNA control from *n* = 4 independent experiments. (**F**) mRNA transcription levels of NLRP3, determined by RT-qPCR. Data represent the fold change as compared to pcDNA control from *n* = 4 independent experiments. All data are indicated as mean ± standard error of the mean. Statistical calculation was performed using unpaired *t*-test related to the pcDNA sample. *P < 0.05, **P < 0.01, ****P < 0.0001. DNP, 2,4-dinitrophenylhydrazone; RLU/s, relative light unit per second; IL-6, interleukin 6; NLRP3, NLR family pyrin domain containing 3 (inflammasome).

Mitochondria represent the main source of reactive oxygen species production with key functions in the overall maintenance of the cellular homeostasis. Notably, the induction of mitochondrial injury and especially respiratory dysfunction promote ROS formation with pleiotropic effects on key proinflammatory mediators (34). Based on this and with respect to the previous observations, we hypothesized a genotype-related induction of oxidative stress and proinflammatory response. To address this question, we performed OxyBlot analysis to determine the oxidative stress level in HBx-transfected Huh7 cells after 72 h p.t. and correlated this with the cellular response reflected by cytoprotective gene expression and activation of proinflammatory mediators, as determined by luciferase reporter assay and RT-qPCR (Fig. 5).

Accordingly, a significant increase of oxidized proteins was observed for those genotypes, which demonstrated in the aforementioned data a high impact on the mitochondrial function/structure, and this is most pronounced not only in GtA but also in GtC (Fig. 5A and B).

However, the strongest induction of NF-кB- and AP-1-dependent promoter activity was observed for HBx-GtB, GtD, and GtE, but not for HBx-GtA, GtC, and GtG as compared to the controlled sample (Fig. 5C and D). This translated to an overall proinflammatory response reflected by a significant increase in interleukin-6 (IL-6) expression in case of GtA, GtE, and GtG, but not GtB, GtC, and GtD (Fig. 5E). Most importantly, the expression of inflammasome subunit NLRP3, a central inflammatory platform after mitochondrial destabilization, is significantly activated in HBx-GtD, GtA, and GtG expressing cells, with the latter two having strikingly strong effects (Fig. 5F). Overall, these data indicate the HBx-dependent impact on mitochondrial integrity induces oxidative stress and proinflammatory response in a genotype-dependent manner. This mainly manifests in the expression of mitochondria-associated inflammasome subunit NLRP3.

### The level of mitophagy is highly influenced by HBx variants

**Fig 6:**
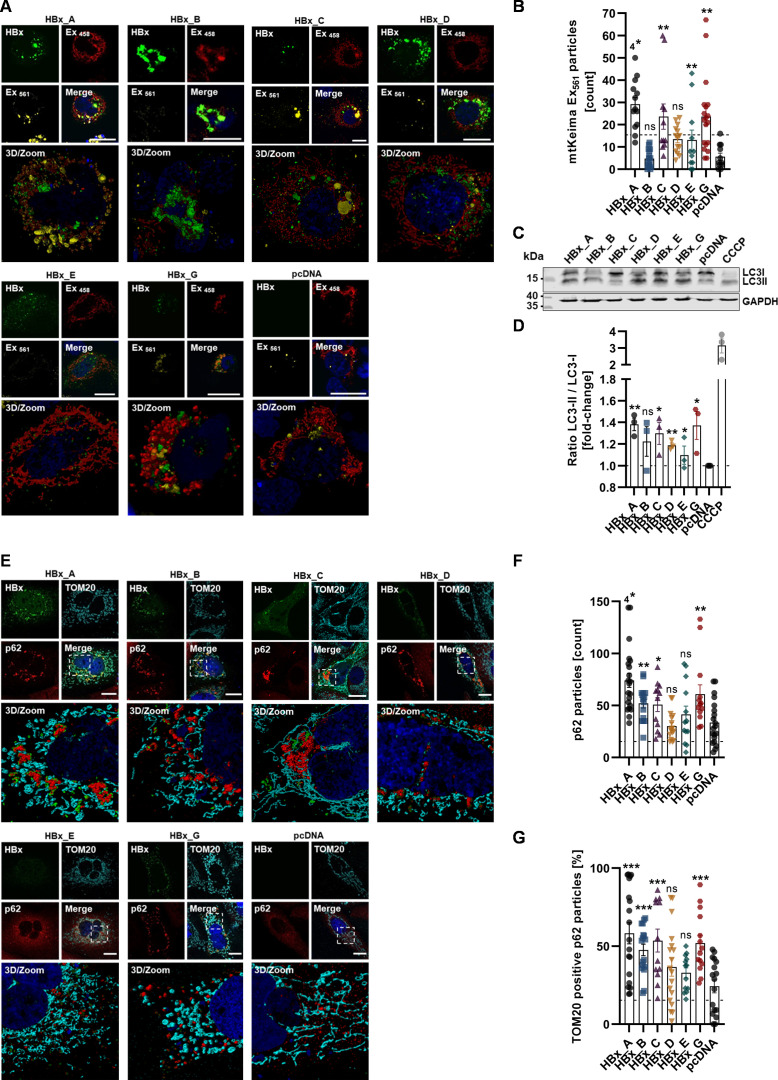
HBx induces mitophagy in genotype-dependent manner. (**A**) Representative confocal microscopy images with 3D high-resolution zoom sections. Cells were co-transfected with the mt-Keima-Red-Mito (red and yellow) plasmid harboring a pH-sensitive fluorophore tagged with a mitochondrial localization sequence and HBx-GFP plasmid (green). Nuclei were counterstained with Hoechst33342 (blue). Scale bar indicates 20 µm. (**B**) Quantification of the average number of acidic mitochondria particles (Ex_561_) per cell. Between 11 and 19 cells per condition were analyzed. (**C**) Representative Western blot of cell lysates, processed for LC3 I and II protein level. Glyceraldehyde-3-phosphate dehydrogenase served as internal loading control. (**D**) Western blot quantification of B; ratio of LC3-II divided by LC3-I was calculated based on *N* = 3 independent experiments. (**E**) Representative confocal microscopy images with 3D-high resolution zoom sections indicated by dashed rectangle. Cells were immunostained for HBx with HA-specific antibody (green), TOM20 as mitochondrial marker (cyan) and p62 (red). Nuclei were counterstained with DAPI (blue). Scale bar indicates 20 µm. (**F**) Quantification of the average number of p62-positive particles per cell in E. Between 14 and 21 cells per condition were analyzed. (**G**) Quantification of TOM20-positive p62 particles as percentage of total p62 particles per cell. More than 14 cells per condition were analyzed. Data are the mean ± standard error of the mean. Statistical analysis was performed using unpaired *t*-test related to control. *P  <  0.05; **P  <  0.01; ***P  <  0.001; ****P  <  0.0001.

In response to elevated mitochondrial injury, cells attempt to maintain the cellular energy homeostasis by a rapid removal of damaged mitochondria, called mitophagy (35). In light of this, we studied the impact of the genotype-dependent effect of HBx on mitophagy using a mtKeima-based mitophagy reporter assay, which was analyzed by quantitative CLSM analysis. Additionally, this was correlated with the amount of mitochondria being associated to p62 and the upregulation of LC3-II. Both are autophagic markers, which are involved in the organelle-specific degradation process, as depicted in Fig. 6.

Again, an overall induction of mitophagy could be observed for HBx-expressing cells. This was most pronounced in the case of HBx of GtA, GtC, and GtG, as evidenced by a dramatic shift in mtKeima spectral features. For GtB and GtE, a non-significant increase was observed (Fig. 6A and B). To further validate this, the autophagy marker proteins LC3I/LC3II were determined by Western blot analysis. Accordingly, similar genotype-related results as before were obtained, with HBx from GtA, GtC, and GtG leading to the greatest increase in LC3I/LC3II ratios (Fig. 6C and D). This was found to coincide with an increased p62 amount as well as an increased co-localization between TOM20 and p62, especially for GtA, GtC, and GtG (Fig. 6E through G). Taken together, these observations indicate that HBx-dependent induction of mitophagy and mitochondrial engulfment in autophagosomal vesicles strongly depend on the genotype, with a dominant effect of the HBx genotypes A, C and G.

### HBx genotypes affect mitophagy through PINK1/Parkin-mediated signaling in a genotype-dependent manner

**Fig 7:**
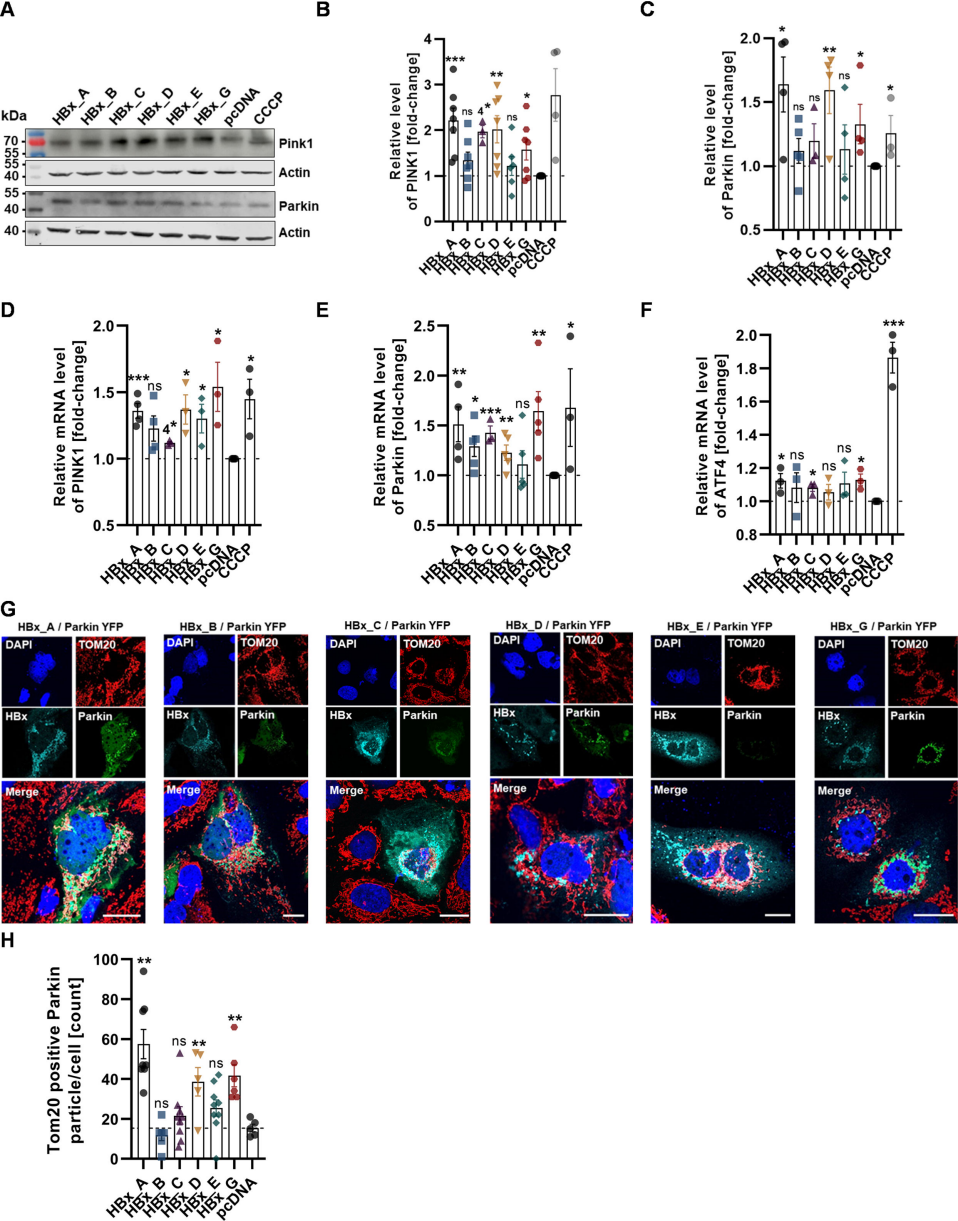
HBx induces genotype-dependent PINK1/Parkin-mediated mitophagy. Huh7 cells were transiently transfected with HBx plasmid DNA of either GtA, GtB, GtC, GtD, GtE, GtG or empty plasmid DNA as control, and fixed for immunofluorescence or harvested 72 h p.t. (**A**) Representative Western blot analysis of PINK1 and Parkin protein levels. β-Actin was used as internal loading control. (**B**) Western blot quantification of panel A; relative PINK1 protein level was calculated based on *n* = 7 independent experiments. (**C**) Western blot quantification of panel A; relative Parkin protein level was calculated based on *n* > 3 independent experiments. (**D**) mRNA transcription levels of PINK1, determined by RT-qPCR. Data represent the fold change as compared to pcDNA control from *n* > 3 independent experiments. (**E**) mRNA transcription levels of Parkin, determined by RT-qPCR. Data represent the fold change as compared to pcDNA control from *n* > 3 independent experiments. (**F**) mRNA transcription levels of ATF4, determined by RT-qPCR. Data represent the fold change as compared to pcDNA control from *n* > 3 independent experiments. (**G**) Representative confocal microscopy images. Cells were co-transfected with a yellow fluorescent protein (YFP)-tagged Parkin plasmid (green) and HBx-HA plasmid. Cells were immunostained for HBx with HA-specific antibody (cyan) and TOM20 as mitochondrial marker (red). Nuclei were counterstained with DAPI (blue). Scale bar indicates 20 µm. (**H**) Quantification of TOM20-positive Parkin particles per cell. Between 5 and 10 cells per condition were analyzed. Data are the mean ± standard error of the mean. Statistical analysis was performed using unpaired *t*-test related to control. *P < 0.05, **P < 0.01, ***P < 0.001, ****P  <  0.0001.

Mitophagy and guidance of damaged mitochondria toward the lysosomal system can be mediated by the PINK1. PINK1 is located at the outer mitochondrial membrane and is rapidly internalized in case of healthy conditions. Defective mitochondria initiate the stabilization of PINK1 and recruit the E3 ubiquitin ligase Parkin on the outer mitochondrial membrane, which activates mitochondrial selective degradation (35, 36). Hence, to understand the genotype-related mechanisms of mitophagy induction in response to elevated HBx levels, we determined levels of PINK1 and Parkin by Western blot and RT-qPCR. Recruitment of Parkin toward mitochondria was monitored by confocal microscopy (Fig. 7).

Overall, based on our data, the presence of HBx leads to an increased level of PINK1- and Parkin-specific transcripts, as well as to an increase in respective protein amount (Fig. 7A through E). Interestingly, higher PINK1/Parkin transcript and protein levels were detected for those HBx variants, with previously observed severe mitochondrial damage and mitophagy. Especially for HBx-GtA, GtC, and GtG, an over 1.5-fold increase in the PINK1 protein amount was detected compared to the control. HBx-GtB and GtE have no significant change compared to the control (Fig. 7A through C). In this regard, a key regulator in the mitochondrial stress response is represented by the transcription factor ATF4 (37). The mRNA level of ATF4 is elevated for HBx-GTA, GtC, and GtG compared to the control but not for the other analyzed genotypes (Fig. 7F). Similarly, amount and localization of Parkin, as evidenced by confocal immunofluorescence images, could barely be visualized for the control cells (reflect intact mitochondria), and there was also only a weak signal in case of HBx-GtB and GtE. For the other tested genotypes, a strong induction in the overall Parkin fluorescence signal intensity and a perinuclear accumulation with clear co-localization to the outer mitochondrial membrane were visible (Fig. 7G). A quantification of TOM20 containing Parkin puncta per cell confirmed these observations (Fig. 7H). In summary, these data indicate that HBx affects mitophagy through PINK1/Parkin-mediated signaling in a genotype-dependent manner, in particular with major effects for the genotypes A, C, D, and G.

### Genotype-specific changes are visible in the context of a full-length HBV replicon system

**Fig 8:**
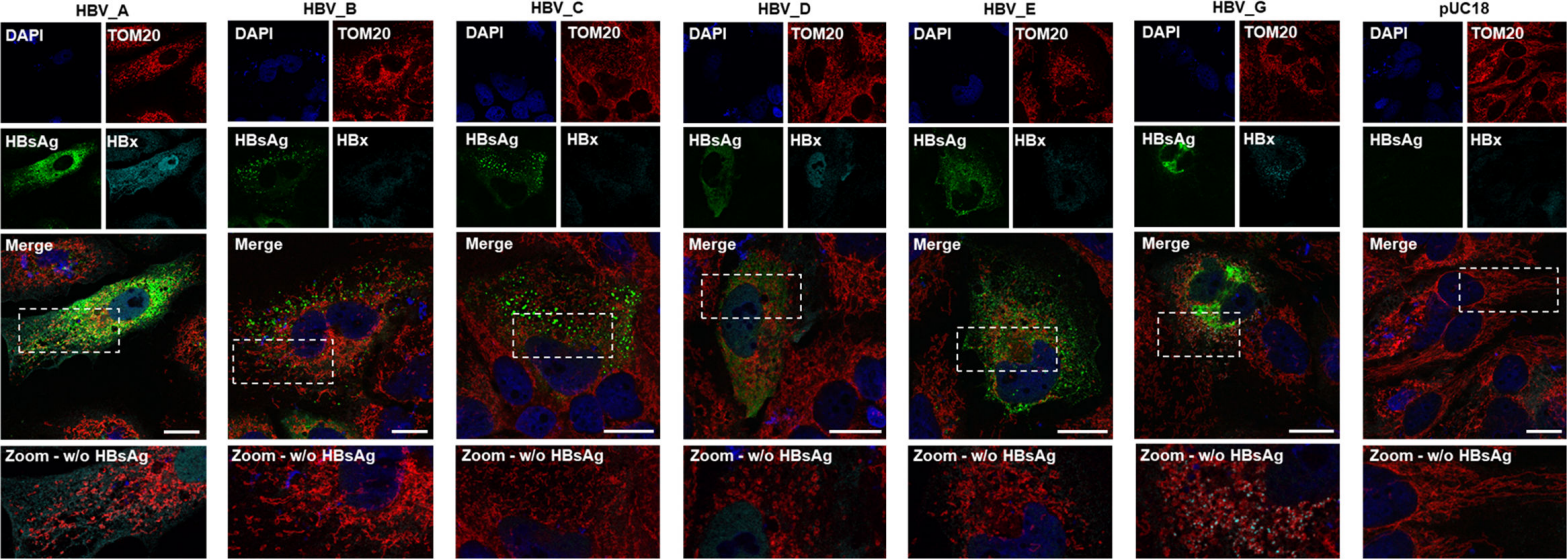
Genotype-dependent changes in mitochondrial dynamics are observable in the presence of complete viral genome replication. Huh7 cells were transiently transfected with 1.3× HBV genomes under autologous promotor control of either GtA, GtB, GtC, GtD, GtE, GtG or empty plasmid DNA as control, and fixed for immunofluorescence 72 h p.t. Representative confocal microscopy images immunostained for HBsAg (green), HBx (cyan), and TOM20 antibody (red). Nuclei were counterstained with DAPI (blue). Scale bar indicates 20 µm. Zoom sections were indicated by dashed rectangles and without HBsAg staining. Scale bar indicates 20 µm.

To examine the influence of HBx-mediated and genotype-dependent effects on mitochondrial structure in the presence of the entire viral life cycle, we performed immunofluorescence microscopy on Huh7 cells, which were transiently transfected with 1.3× HBV replicon constructs of the different genotypes. Since in this study design we were not able to properly detect the viral HBx protein, we additionally used an HBsAg-specific antibody to visualize HBV-positive cells in the setting. All HBV-positive cells, independent of the genotype, demonstrate a higher rate of fragmentation as compared to the pUC18-transfected control. However, it can be seen that the condensed and perinuclear accumulation of mitochondria, as previously observed in the HBx-transfected cells, is not induced in case of HBV-transfected cells. Clear changes in the tubular structures are particularly visible not only in HBV-GtA but also in GtC and GtD (Fig. 8).

## DISCUSSION

In the past years, more attention was given to the relationship between the genetic diversity of HBV and the modulation of virus-associated pathogenesis. The focus was on the genotype-specific pathogenesis and treatment response. Nonetheless, detailed *in vitro* studies especially with a direct comparison of different HBV genotypes are heavily underrepresented but are considered as an essential aspect for the development of an effective and specific disease management (14, 38).

A pathway enrichment analysis of HBx-GtA indicated profound levels of several deregulated kinase pathways. Notably, a remarkable number of upregulated kinases were found to be associated with mitochondrial functions as well as oxidative stress-related pathways. Based on these findings, a closer comparative analysis of the different HBx genotypes indicated prominent differences in the kinome profile, especially with respect to the deregulation of mitochondrion-specific kinases. As a central example in our study, members of the Src-family kinases, which are heavily involved in the modulation of respiratory function as well as mtROS-mediated activation of the NF-кB pathway (33, 39), were significantly upregulated only in some of the tested HBx genotypes. Similar results were also obtained for other kinases such as AKT, ErbB2, or JNK, which are related to mitochondrial function. Based on this, it was speculated that the genetic variants of HBx could differ especially in mitochondrion-associated pathways, mitophagy, and processes occurring in response to oxidative challenge.

Indeed, the sophisticated system of mitochondrial dynamics is in general a common target for several viral proteins from a variety of different virus types, including HBV (23, 40, 41). Manipulation of the “housekeeping” functions of mitochondria can affect viral replication and escape from the host anti-viral immune response (42). There was evidence that HBx affects mitochondrial structure and thereby could cause profound liver-pathological consequences; however, the impact of the genotype was unclear. Fragmentation of the overall mitochondrial architecture (mitochondrial fission) is considered as initial step in order to remove non-functional mitochondrial structures and initiate a cascade of reactions. This includes mitochondrial quantity control mechanisms, elimination of defective mitochondria accompanied by a reduction of the mitochondrial mass, alongside with and induction of cellular ROS levels, followed by an elevated inflammatory response (26, 31, 43).

In accordance to our kinome data, we found profound differences in the mitochondrial network integrity among the genetic variants of HBx. Beyond, HBx from all tested genotypes led to a reduction in the overall mitochondrial footprint. Interestingly, this effect is most pronounced for genotypes A, D, and G; especially HBx from GtA and GtG completely abolished the network-like structure of the organelles, in combination with a clear reduction in the overall mitochondrial protein mass. This observation speaks for a conversion of the long filamentous and branched structures, which were observed in the control samples and indicate a healthy cellular physiology, into small round fragments as response to elevated mitochondrial dysfunction. It can be assumed that the reduction in the mitochondrial mass, prominently observed for the HBx variants A and G, is due to the perturbation of mitochondrial fission accompanied by an elevated removal process of non-functional mitochondria.

Depolarized mitochondria are a target for mitophagy. Accordingly, to these findings described above, we also observed distinct expression levels of the mitophagy-mediating genes *PINK1/Parkin* and an accumulation of Parkin along mitochondria, depending on the genotype of HBx. Therefore, the PINK1/Parkin pathway is the suggested route to induce mitophagy in HBx-transfected cells. Indeed, genotypes B and E showed a significantly lower influence on the mitochondrial functions and structures and, in accordance to this, less PINK1/Parkin-mediated mitophagy. This may be due to the moderate number of fragmented mitochondria and overall lower mitochondrial injury. In this context, the transcription factor ATF is known as a key affecter in response to mitochondrial stress. On the one hand, activated ATF reduces the activity of mitochondria, to prevent elevated ROS production; on the other hand, it coordinates the mitochondrial stress response by mediating the upregulation of Parkin. In addition, ATF also activates cytoprotective gene expression and inflammatory responses. Accordingly, significant upregulation of the ATF4 mRNA levels as observed for HBx-GtA, GtC, and GtG might be reasonable for elevated mitophagy and activation of PINK1/Parkin signaling in response to elevated ROS level but also explain the increase in inflammatory signaling, especially in these genotypes.

Indeed, HBV genotypes differ with respect to the HBx-dependent impact on the intracellular ROS level. In particular, HBx-GtA and GtG are accordingly associated with an elevated cellular ROS level. Hence, the expression of the NLRP3 inflammasome, a key regulatory platform for mitochondrial inflammatory processes (44), as well as IL-6 as representative of proinflammatory mediators (32), was highly upregulated by these genotypes. As mitochondrial integrity was only slightly compromised for HBx-GtB and GtE, we hypothesized about corresponding minor effects on oxidative stress levels and associated signaling pathways. Our data confirm this in case of GtB. Interestingly, however, GtE showed a noticeably different effect with a significant increased ROS level and elevated IL-6 and NLRP3 expression. However, it should be considered that, on the one hand, there is the process of mitochondria-dependent formation of ROS, and on the other hand, there is the modulation of ROS-detoxifying pathways as the Nrf2/ARE-dependent induction of cytoprotective genes. The actual ROS-level reflects the interplay of a variety of processes, so additional studies may focus on disambiguating these relations.

To provide further insights into the mitochondrial function, we determined the interaction of the HBx protein with the mitochondrion-associated VDAC3. In particular, it has recently been described that HBx interacts with VDAC3 and thereby impairs mitochondrial functions, leading to elevated ROS levels (29, 34). Our data demonstrate now a clear divergence among the genetic HBx variants. Moderate levels of VDAC3-HBx colocalization were observed for HBx-GtB and GtE, accompanied by a moderate impact on the membrane potential. Interestingly, in contrast to this, for HBx-GtA and GtG, an increased level of colocalization between the human VDAC3 and the HBx protein and a significant decrease in mitochondrial membrane potential were observed. These data evidence a genotype-dependent contribution of HBx-related mitochondrial dysfunction. Moreover, we investigated the activity of the cytochrome c oxidase, which is part of the respiratory electron transport chain and is thus central for the ATP synthesis and maintenance of the metabolic homeostasis (45). Here, we observed that the HBx protein in general reduces the cytochrome c oxidase activity but with a significant decline for HBx of genotypes G, D, and B. Despite the finding that the high standard deviations within the cytochrome c oxidase activity assay hamper a precise genotype-related investigation, the overall trend corresponds to the other observations.

Overall, these data indicate a functional but genotype-specific crosstalk between HBx and mitochondrion-associated mechanisms. In particular, our findings provide first-time evidence that the HBx of genotypes A and G heavily impairs the mitochondrial network structure, which occurs in response to elevated HBx-mediated mitochondrial dysfunction. However, genotypes B and E of HBx induce apparently only a moderate effect in this regard. Apparently, HBx-GtC has moderate effects in the mitochondrial structures but highly affects the mitochondrial function and induces mitochondrial function and ROS generation, with related effect on the mitochondrial stress response system.

**Fig 9:**
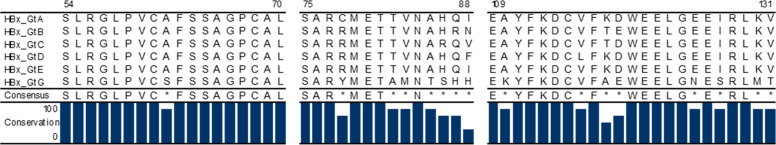
Sequence comparison of amino acids 54–70, 75–88, and 109–131 in the HBx of different genotypes. Residues in these sequences represent the predicted mitochondrial interaction sites of HBx.

Although the compartmentalization and also the interaction of HBx with mitochondrial proteins are still under debate and controversy discussed, HBx has been previously shown to interact with the human VDAC and to affect functionality (29). In this regard, it should be noted that there are in total three mitochondrial interaction sites in the HBx sequence. While the N-terminal region (aa 54–70) is highly conserved among the different genotypes, the two C-terminal mitochondrial binding sites (aa 75–88 and aa 109–131) (38) are located within the transactivator domain of the HBx amino acid sequence and are highly heterogeneous among the different HBV genotypes. Importantly, these sited were previously described to be essential for the mitochondrial localization; cysteine-115 was especially identified as key aa for the mitochondrial interaction (46) and is conserved in all of our studied genotypes (Fig. 9). This may cause the interaction among all genotypes in our study. However, the heterogenous appearance in the other aa of the mitochondrial binding site may be reasonable for the different effects on mitochondrial function but needs further extensive investigations to confirm this assumption and is outside of the scope of the current work. Furthermore, a distinct solubility among HBx genotypes due to different sequence motives might lead to different cellular aggregation states and heterogeneity in its cellular distribution, and affects its functional properties and ability to modulate cellular processes. This could also explain the observed heterogeneous distribution and partially punctate structure in some of the studied genotypes.

In summary, based on our data, we provide direct evidence that HBx-mediated changes in the mitochondria morphology and function are fundamentally influenced by the different genotypes. In particular GtA and GtG, followed up by GtC and GtD, heavily alter essential components in the mitochondrial dynamic, together with a clear involvement in oxidative stress related processes and inflammation response. Contrary to that, GtE and also GtB cause very moderate changes and seems to retain mostly the overall mitochondrial physiology. These data do not directly allow a correlation between impact on the mitochondrial function and integrity on the one hand and outcome of the chronic infection on the other hand. This might reflect that HBV-associated pathogenesis is the result of a multifactorial process encompassing host factors and further viral regulatory proteins as the PreS2 activators.

On the one hand, the lack of these additional viral factors represents a limitation of our study; on the other hand, it was the explicit aim of the study to investigate the HBx-specific effect, depending on the HBV genotype in the absence of further interfering viral proteins. For this purpose, the selective transient overexpression of HBx of a different genotype system was chosen to elucidate these questions. An initial experiment with expression of the entire viral genome also showed visible structural changes, particularly in the genotypes with extreme mitochondrial damage during HBx overexpression. Genotypes B and E have only a minor visible impact on this result, which could probably be due to correlating or overlapping effects of the other viral proteins on host-signaling pathways. In light of the strong differences concerning the geographic distribution of the genotypes, host factors and further factors such as lifestyle and nutrition are additional relevant variables affecting the outcome of chronic infection. With focus on the main genotype-related effects of HBx on key regulatory pathways of the host, this work contributes to the mechanistic understanding of HBx-mediated liver pathogenesis, depending on the genotype, and thereby deepens the knowledge about the impact of the genotype on the outcome of HBV infection contribution of HBV.

## MATERIALS AND METHODS

### Cell culture and treatment

The human hepatoma cell line Huh7 (47) and HepG2 were maintained in either Dulbecco’s Modified Eagle Medium (DMEM) (Huh7 cells) or Roswell Park Memorial Institute Medium-1640 (HepG2) supplemented 10% (vol/vol) fetal calf serum (Bio & Sell, FBS.S0615), 100-units/mL penicillin, 100-µg/mL streptomycin, and 2-mM L-glutamine and cultured in a humidified atmosphere of 5% CO_2_ and 37°C. As control for mitochondrial dysfunction, cells were exposed to 10-µM CCCP (Invitrogen) 12 h prior to harvesting.

### Plasmid construction and transfection

HBx plasmids with C-terminal human influenza HA tag were generated using restriction cloning. X-ORFs of GtA (GenBank: OR580945.1^1376–1837^), GtB (GenBank: OR580947.1^1376–1837^), GtC (GenBank: AB675679.1^1374–1836^), GtD (GenBank: OR580949.1^1376–1837^), GtE (GenBank: MN172189.1^2788–3250^), and GtG (GenBank: DQ207798.1^1371–1832^) were amplified from respective genotypes of HBV replicon plasmids by PCR (using Q5 polymerase, New England Biolabs, M0491), prior to restriction digestion with HindIII-HF and NheI-HF (New England Biolabs, #R3104 and #R3131) and ligation into pcDNA3.0 vector (Invitrogen) in-frame with an HA tag. HBx-GtA (no tag) was cloned accordingly but without the N-terminal tag in the restriction primer. HBx-eGFP expression constructs were performed with Gibson assembly method. In brief the X-ORF of GtA, GtB, GtC, GtD, GtE and GtG as well as an eGFP coding region were PCR-amplified with 20- to 40-bp homologous sequences overlap between the respective neighbor fragments by using Q5 polymerase. PCR fragments were gel extracted (Qiagen, #28704) and Gibson assembled into EcoRV-HF pre-digested pcDNA3.1(-) backbone.

Replication competent HBV plasmids carrying 1.3-fold autologous promotor-driven HBV genomes of genotypes A, B, C, D, E, and G were cloned into a pUC18 vector as described previously (48). Briefly, the full-length HBV genome was amplified by PCR using primers P1 and P2 with Q5 polymerase. The 3.2-kb amplicon was SapI digested and gel extracted prior to self-ligation to 6.4-kb fragments. Fragments A and B were amplified from 2× HBV genome by PCR prior restriction digestion with either HindIII-HF and XbaI (Fragment A) or XbaI and BglII (Fragment B). The digested fragments were ligated into Bam-HI and HindIII-HF pre-digested pUC18 backbone.

All primers used for cloning were synthesized by Eurofins Genomics and indicated in Table 1.

**TABLE 1:** Primer sequences used for cloning

Name	Sequence
HBx_gtA_NheI_fw	5′-CTA GCT AGC ATG GCT GCT AGG CTG TAC TGC-3′
HBx_gtB/D/G/E_NheI_fw	5′-CTA GCT AGC ATG GCT GCT AGG CTG TGC TGC-3′
HBx_gtA/B/D/E_HA_Hindlll_rev	5′-CCC AAG CTT TTA GGC ATA ATC TGG CAC ATC ATA AGG GTA GGC AGA GGT GAA AAA GTT GCA TG-3′
HBx_gtG_HA_HindlIl_rev	5′-CCC AAG CTT TTA GGC ATA ATC TGG CAC ATC ATA AGG GTA GGC AGA GGT GAA AAA GTT ACA TG-3′
HBx_gtA_(no tag)_HindIII_rev	5′-CCC AAG CTT TTA GGC AGA GGT GAA AAA GTT GCA TG-3′
HBx_fw_fusion primer	5′-TCG AGC GGC CGC CAC TGT GCT GG A TAT GGC TGC TAG GCT GTA TGC-3′
HBx_gtC_fw_fusion primer	5′-TCG AGC GGC CGC CAC TGT GCT GGA TAT GGC TGC TAG GGT GTG CTG C-3′
HBx_rev_fusion primer	5′-GGA TCC TCC GGA TCC TCC GAT ATC GGC AGA GGT GAA AAA GTT GCA TG-3′
HBx_gtD_rev_ fusion primer	5′-GGA TCC TCC GGA TCC TCC GAT ATC GGC AGA GGT GAA AAA GTG GCA AG-3′
HBx_gtG_rev_ fusion primer	5′-GGA TCC TCC GGA TCC TCC GAT ATC GGC AGA GGT GAA AAA GTT ACA TG-3′
eGFP_fw_fusion primer	5′-GAT ATC GGA GGA TCC GGA GGA TCC GTG AGC AAG GGC GAG GAG-3′
eGFP_rev_fusion primer	5′-TCC AGT GTG GTG GAA TTC TGC AGA TTT ACT TGT ACA GCT CGT CCA TGC-3′
P1 (full-length HBV)_fw	5′-CCG GAA AGC TTG AGC TCT TCT TTT TCA CCT CTG CCT AAT CA-3′
P2 (full-length HBV)_rev	5′-CCG GAG TCG ACG AGC TCT TCA AAA AGT TGC ATG GTG CTG G-3′
Fragment_A_(HBV)_fw	5′-CCC AAG CTT CTA TTG ATT GGA AAG TAT GTC-3′
Fragment_A_(HBV)_rev	5′-GAA AAT TGA GAG AAG TCC AC-3′
Fragment_B_(HBV)_fw	5′-ACA ARA ATC CTC ACA ATA CC-3′
Fragment_B_(HBV)_rev	5′-GAA GAT CTG ATA GGG GCA TTT GGT GGT C-3′

Plasmids for NF-kB and AP-1 reporter gene assay contain a luciferase reporter construct in frame with the antioxidant response elements of the target gene. The NF-кB luciferase reporter plasmid was purchased from GenScript. The AP-1 luciferase reporter was described in Hildt et al. (49). pM-Keima-Red-Mito was a gift from Michael Davidson (Addgene, #56018); Parkin-YFP was a gift from Richard Youle (Addgene, #23955) (50). All plasmids were prepared and purified using Maxi-prep kit (Qiagen) and verified by DNA sequencing.

Fugene DNA transfection reagent was employed for transient transfection or plasmid co-transfection of Huh7 cells by using a 3:1 ratio according to the manufacturer’s instructions, except for an additional medium exchange, 16 h post transfection. Transfection with pcDNA plasmid DNA served as an empty vector control. Cells were usually harvested 72 h after transfection, resulting in a routinely obtained transfection effectiveness of 40%–55%, based on flow cytometry analysis.

### SDS-PAGE and Western blotting

Total protein was extracted with radioimmunoprecipitation assay (RIPA) lysis buffer [50-mM Tris, 150-mM NaCl, 0.1% (wt/vol) SDS, 0.5% (wt/vol) sodium desoxycholate, 1% (vol/vol) Triton X-100, pH 7.2], including protease inhibitors, and was separated by SDS-polyacrylamide gel electrophoresis followed by transfer onto polyvinylidine fluoride (PVDF) membranes according standard protocol (51). Membranes were incubated with primary antibody against glyceraldehyde-3-phosphate dehydrogenase (Santa Cruz, #sc-47724), MtCOX2 (Santa Cruz, #sc-514489), COX4 (Santa Cruz, #sc-376731), HA tag (Thermo Scientific, #26183), LC3B (Biozol, #PM036), and PINK1 (Cell Signaling, #6946), Parkin (Cell Signaling, # 2132), TOM20 (Santa Cruz, #sc-17764), green fluorescent protein (Invitrogen, #A-11122), HBx (Invitrogen, #MA1-081), or β-actin (Sigma-Aldrich, #A5316) overnight at 4°C. Detection of primary antibodies was performed by using either a horseradish peroxidase (HRP)-conjugated secondary antibody (Cytiva, #GENA934) and ECL Detection reagent (Immobilon Forte Western HRP-Substrat, Millipore, #WBLUF) with the Amersham ImageQuant800 Imaging system (Cytiva), or the LI-COR Odyssey infrared imager (LI-COR Biosciences) together with the provided secondary antibody of the company. Protein band intensities were analyzed by Studio Light Imaging software (v.5.2, LI-COR Biosciences) and normalized to a respective reference protein.

### Reactive oxygen species detection

Carbonylated proteins were investigated using OxyBlot protein oxidation detection kit (Merck Millipore, #S7150) according to the manufacturer`s instructions. Anti-β-actin (Sigma-Aldrich, #A5316) was used as loading control.

### Luciferase reporter gene activity assay

Cells were transiently co-transfected with the indicated luciferase reporter gene plasmid DNA and respective HBx plasmid and harvested 48 h post transfection. Cells were lysed by adding luciferase lysis buffer [25 mM Tris-HCl, 2 mM dithiothreitol (DTT), 2 mM ethylene glycol tetraacetic acid (EGTA), 10% (vol/vol) glycerol, 0.1% (vol/vol) Triton X-100, pH 7.5]. Measurement of the chemiluminescence was performed in an Orion II microplate luminometer (Berthold Detection Systems) using luciferase substrate (20 mM Tris-HCl, 5 mM MgCl_2_, 0.1 mM EDTA, 33.3 mM DTT, 470 µM d-luciferin, 530 µM ATP, pH 7.8). Relative light intensities per second were normalized to the total protein amount using Bradford assay according to the manufacturer’s protocol. The data were calculated as normalized fold change compared to control pcDNA.

### RNA extraction and real-time qPCR

Intracellular RNA was isolated using RNA-Solv Reagent (Omego Bio-Tek, #R6830) and reverse transcribed into complementary DNA, applying the RevertAid H minus RT Kit (Thermo Scientific, #EP0452). Subsequently, quantitative real-time PCR for specific transcripts was carried out with the Maxima SYBR-Green qPCR Kit (Thermo Scientific, #K0221). The housekeeping gene hRPL27 was used as internal control. All gene-specific primers used in this study are listed in Table 2. Samples were measured in duplicate with the LightCycler 480 Instrument II (Roche) and analyzed as fold-change values as compared to the control, achieved by applying the ΔΔC_T_ method (52).

**TABLE 2:** Primer sequences used for qPCR analyses

Name	Sequence
Human-RPL27_fw	5′-AAA GCT GTC ATC GTG AAG AAC-3′
Human-RPL27_rev	5′-GCT GCT ACT TTG CGG GGG TAG-3′
Human_NLRP3_fw	5′-GGT GGA GTG TCG GAG AAG-3′ (53)
Human_NLRP3_rev	5′-CTG TCA TTG TCC TGG TGT CT-3′ (53)
Human_TOMM20_fw	5′-TTT GGG GAG GGT AGA AAC GC-3′ (54)
Human_TOMM20_rev	5′-TTC AGA GCC AAG TGA CAC CC-3′ (54)
Human_IL6_fw	5′-GGA GAC TTG CCT GGT GAA AAT CAT CAC-3′
Human_IL6_rev	5′-AGC AGG CTG GCA TTT GTG GTT G-3′
Human_TNFα_fw	5′-GTT CCT CAG CCT CTT CTC CTT CCT G-3′
Human_TNFα_rev	5′-ACA ACA TGG GCT ACA GGC TTG TCA C-3′
Human_IL1β_fw	5′-GAG CTC GCC AGT GAA ATG ATG-3′
Human_IL1β_rev	5′-TAG TGG TGG TCG GAG ATT CG-3′
Human_PTEN induced kinase 1_fw	5′-GGC CTT GGC TGG GGA GTA TG-3′
Human_PTEN induced kinase 1_rev	5′-GCG GAG AAC CCG GAT GAT GT-3′
Human_Parkin_fw	5′-GTG TTT GTC AGG TTC AAC TCC A-3′
Human_Parkin_rev	5′-GAA AAT CAC ACG CAA CTG GTC-3′
Human_ATF4_fw	5’- CCC TTC ACC TTC TTA CAA CC TC-3′ (55)
Human_ATF4_rev	5’- GTC TGG CTT CCT ATC TCC TTC A-3′ (55)

### Immunofluorescence microscopy

Cells were grown on glass coverslips and fixed 72 h post transfection with 4% formaldehyde in phosphate-buffered saline (PBS) (Carl Roth, #CP10). Subsequently, cells were permeabilized with 0.5% Tween 20 in PBS; blocking was carried out with 5% (wt/vol) bovine serum albumin (Carl Roth, T844) in PBS, followed by incubation with respective primary antibody against p62 (ProGen, #GP62-C), HA tag (Thermo Scientific, #26183; Cell Signaling, #3724; Novus, #NB600-362), TOM20 (Santa Cruz, #sc-17764; Abcam ab186735), HBx (Invitrogen, #MA1-081), or VDAC3 (Thermo Scientific, PA5-87973). Samples were then incubated with the respective secondary antibody conjugated with either AlexaFluor488, 546, or 633 coupled donkey-anti-mouse, anti-rabbit, anti-goat, or anti-guinea pig IgG (Invitrogen) or HBsAg-FITC (Abcam, #ab32914). Phalloidin-Atto 633 (Sigma-Aldrich) was used for F-actin visualization; the nuclei were stained with 4′,6-diamidin-2-phenylindole (250 ng/mL, Carl Roth, #6335). Mowiol-mounted cells were imaged on a confocal laser scanning microscope, Leica TCS SP8 System with a DMi8 microscope (Leica), using a ×100 magnification oil objective (numerical aperture = 1.4), at 1 airy unit, resulting in an optical thickness of 0.7 µm. Image acquisition, as well as deconvolution using the lightning tool application, was performed with the LasX control software (Leica). Single-cell analysis of the CTCF and tMOC, as well as quantification of double-positive particles, was investigated with FIJI software (open source) (56).

### Mitochondria network analysis

Mitochondrial network structures were processed with the Mitochondrial Network Analysis tool (MiNa) in FIJI, as described previously by Valente et al. (57). The source code was made available by the programmer in the GitHub repository and was installed accordingly (58). Immunofluorescence-stained samples were imaged by confocal microscopy, and mitochondrial structures stained for the mitochondrial outer membrane marker protein (Tom20) in HBx-positive cells were quantified using MiNa analysis tool. Application of the tool provides an overlay of a binarized mitochondria and skeletal model used to determine the mitochondrial footprint (reflects the area consumed by mitochondrial pixels) and the mean network count (indicates the mean number of connected lines reflecting the bifurcation of the mitochondrial network). A minimum of 12 cells of each construct were used for statistical analysis.

### Mt-mKeima mitophagy assay

As reporter assay for mitophagy in living cells via mt-Keima, Huh7 cells were cultured in IBIDI chamber slides (IBIDI, #80286) and were transiently co-transfected with the respective HBx-eGFP plasmid, together with pM-Keima-Red-Mito plasmid (Addgene, #56018) for 72 h. Prior to imaging, nuclei were stained with Hoechst33342 (Life Technologies, #H3570) diluted in culture medium for 10 min at 37°C. Subsequently, cells were washed twice with PBS and maintained in FluoroBrite DMEM medium (Gibco, A1896701). Images were examined on a Leica SP8 confocal microscope using a ×63 magnification objective (numerical aperture = 1.4). Fluorescence of mt-Keima was imaged in two channels via sequential excitations at 458 nm for mtKeima in “neutral” mitochondria and at 561 nm for mtKeima in acidic mito-lysosomes. The emission range was set between 570 and 690 nm. HBx-positive cells were identified by eGFP signal using a 488-nm excitation with a 495- to 550-nm emission filter. Double-positive cells were imaged using the LasX control software (Leica).

### Mitochondria membrane potential

The MitoProbe JC-1 Assay-Kit (Thermo Scientific, M34152) was performed according to the manufacturer’s instruction to determine mitochondrial membrane potential in each sample. In brief, transiently transfected Huh7 or HepG2 cells were collected 72 h post transfection by using Accutase (Merck Millipore, SCR005) and washed twice with PBS. Cells (1 × 10^6^) were resuspended in 1-mL JC-1 dye diluted in PBS (final concentration 2 µM) and incubated for 20 min at 37°C in the dark. Samples were washed twice with PBS before fluorescence intensity was measured using a MACSQuant10 flow cytometer (Miltenyi) with 488-nm excitation and 529- and 590-nm emission filters. A total amount of 1 × 10^4^ cells per sample were recorded and analyzed using FlowJo software (v.10, FlowJo, LCC). The ratio between red (JC-1 aggregates) to green (JC-1 monomers) mean fluorescence intensities was calculated and normalized to control sample.

### Complex IV activity assay

For complex IV activity assay, mitochondria were isolated from transiently transfected cells 72 h post transfection using the commercial Mitochondria Isolation Kit for Cultured Cells (Thermo Scientific, 89874) according to the manufacturer’s instructions. Approximately 1 × 10^7^ cells were trypsinized and washed twice with PBS, followed by detergent-based method and centrifugation at 6,000 × g for 15 min. A total amount of 10-µg mitochondria was subjected to complex IV activity measurement, which was performed with the Complex IV Human Specific Activity Microplate Assay Kit (Abcam, ab109910). The complex IV activity assay is based on the immobilization of cytochrome c oxidase in the assay plate and determination of activity by monitoring a decreased absorbance at 550 nm over time due to oxidation of the reduced cytochrome c substrate. Subsequently, the rate of enzyme activity was calculated within the linear range and correlated to the control sample.

### Kinome profiling

Transiently transfected cells were harvested 72 h post transfection by adding M-PER Mammalian Protein Extraction Reagent (Thermo Scientific, 78503), including Halt Protease Inhibitor Cocktail (Thermo Scientific, 87785) and Halt Phosphatase Inhibitor Cocktail (Thermo Scientific, 78420). The protein concentration was measured using Pierce BCA Protein Assay Kit (Thermo Scientific, 23225), and equal protein amounts were used to detect phosphorylation status of target-peptides of distinct tyrosine- (PTK) or serine-threonine kinase (STK) of the lysate. STK and PTK PamChip microarrays were loaded and measured with respective reagents kits (PamGene International) using the PamStation (v.12) instrument. Phosphorylation sites of defined peptides, determined by a fluorescent-labeled antibody, were predicted and correlated to upstream kinase activities by using the BioNavigator software (v.6.3.67), as described elsewhere (59). The kinase final score is calculated by the specificity of the peptides corresponding to the kinases and the significance of the changed phosphorylations in the peptides. The kinase activity represents either upregulation or downregulation of the respective kinase, compared to the control. Kyoto Encyclopedia of Genes and Genomes (KEGG) pathway enrichment analysis was performed with KEGG Orthology Based AnnotaHtion System (v.3.0) database tool (60). The gene list of predicted, statistically enriched kinome data, with a mean final score above 1.2, was applied to conduct pathway enrichment analysis by using the default module settings. Enriched terms with a *P* value above 0.05 were visualized in a bubble blot (61).

### Statistical analysis

If not indicated otherwise, all data are expressed as mean value ± standard error of the mean. Experiments were performed in a minimum of three independent approaches. Data analysis was evaluated with GraphPad Prism Software (v.8.0, GraphPad). Data sets presented as fold-change values were calculated for the respective control sample in each independent experiment and were arbitrarily set as 1. For statistical analysis, data sets were analyzed with unpaired, two-tailed Student’s *t*-test. A *P* value of less than 0.05 was considered significant, which is indicated by an asterisk defined by *P < 0.05, **P < 0.01, ***P < 0.001, ****P < 0.0001.

## Data Availability

Raw data of kinome analysis are available in a public repository (Mendeley Data – DOI: 10.17632/3h5r4dwhp2.1).
